# Changing Malaria Epidemiology and Diagnostic Criteria for *Plasmodium falciparum* Clinical Malaria

**DOI:** 10.1371/journal.pone.0046188

**Published:** 2012-09-28

**Authors:** Clémentine Roucher, Christophe Rogier, Fambaye Dieye-Ba, Cheikh Sokhna, Adama Tall, Jean-François Trape

**Affiliations:** 1 Institut de Recherche pour le Développement, Laboratoire de Paludologie, UMR 198, BP 1386, Dakar, Senegal; 2 Institut Pasteur de Dakar, Unité d'Epidémiologie, B.P. 220, Dakar, Senegal; 3 Institut Pasteur de Madagascar, B.P. 1274, 101 Antananarivo, Madagascar; Walter & Eliza Hall Institute, Australia

## Abstract

**Background:**

In tropical Africa, where malaria is highly endemic, low grade infections are asymptomatic and the diagnosis of clinical malaria is usually based on parasite density. Here we investigate how changes in malaria control and endemicity modify diagnostic criteria of *Plasmodium falciparum* attacks.

**Methods and Findings:**

Parasitological and clinical data from the population of Dielmo, Senegal, monitored during 20 years, are analyzed in a random-effect logistic regression model to investigate the relationship between the level of parasitemia and risk of fever. Between 1990 and 2010, *P. falciparum* prevalence in asymptomatic persons declined from 85% to 1% in children 0–3 years and from 34% to 2% in adults ≥50 years. Thresholds levels of parasitemia for attributing fever episodes to malaria decreased by steps in relation to control policies. Using baseline threshold during following periods underestimated *P. falciparum* attacks by 9.8–20.2% in children and 18.9–40.2% in adults. Considering all fever episodes associated with malaria parasites as clinical attacks overestimated *P. falciparum* attacks by 42.2–68.5% in children and 45.9–211.7% in adults.

**Conclusions:**

Malaria control modifies in all age-groups the threshold levels of parasitemia to be used for the assessment of malaria morbidity and to guide therapeutic decisions. Even under declining levels of malaria endemicity, the parasite density method must remain the reference method for distinguishing malaria from other causes of fever and assessing trends in the burden of malaria.

## Introduction

In tropical Africa, where malaria is highly endemic, most individuals are semi-immune and asymptomatic malaria infections are highly prevalent. Thick blood films in children and adults are often positive regardless of the clinical context and the detection of malaria parasites in persons with fever is not sufficient criteria for distinguishing malaria from other causes of fever. Following early studies in the 1980s that have shown that parasitemia was much higher during malaria attacks than during asymptomatic chronic infections and that it was possible to use parasite density thresholds to confirm or to discard the diagnosis of clinical malaria with a low risk of error [1], [2], the parasite density method has become the reference method for measuring malaria morbidity in research and clinical trials in highly endemic areas of tropical Africa [3]–[7].

Numerous studies have been conducted during the two past decades using various approaches and models to investigate the relationship between the dynamics of parasitemia and the occurrence of fever, and to determine levels of *Plasmodium falciparum* parasitemia which constitute the best threshold values for attributing to clinical malaria a fever episode in a given area and population [3]–[14]. These studies have shown that age and endemicity level are the two most important factors that determine these levels. Threshold values are generally much higher in young children than in older children and in adults, and differences between age groups are higher in holoendemic areas than in meso and hypoendemic areas [6]–[8], [10], [13], [14].

Since the mid-2000s, there have been substantial changes in malaria and its control throughout tropical Africa. To face the dramatic consequences of the dissemination of high levels of resistance to chloroquine in *P. falciparum*, all countries gradually abandoned chloroquine, either initially switching to sulfadoxine with pyrimethamine alone or the combination of amodiaquine plus sulfadoxine with pyrimethamine before switching to artemisin-based combination therapy (ACTs), or directly switching to ACTs. In addition, depending on the region, the change in first-line treatment of malaria attacks was preceded, followed, or accompanied by the mass distribution of insecticide-treated bednets [15]–[16]. Studies in several countries have shown that these policies substantially reduced malaria endemicity and the burden of the disease [17]–[20]. This new context of declining malaria in many parts of tropical Africa is likely to have an impact on the rate of acquisition of immunity [21], and thus to modify the levels and dynamics of parasitemia associated with malaria attacks and the accuracy of malaria morbidity estimates based on parasite density.

Since 1990, the population of Dielmo, Senegal, has been involved in a long-term study of the host-vector-parasite relationships in malaria [22]. Daily monitoring of fever and monthly monitoring of asymptomatic parasitemia and malaria transmission have generated a unique dataset, which allows historical analysis of precisely timed interventions on malaria morbidity and epidemiology. In this paper, we examine how changes in first-line treatment of malaria attacks and the introduction of long-lasting insecticide-treated nets (LLINs) has modified parasite prevalence and density at the community level, and how it has affected diagnostic criteria for *P. falciparum* malaria attacks based on parasite density measurement.

## Population and Methods

### Study area and participants

The study was carried out in Dielmo, a village situated in a Sudan-savannah region of central Senegal, on the marshy bank of a small permanent stream, where anopheline mosquitoes breed all year round [22]. Malaria transmission is intense and perennial, with a mean 258 and 132 infected bites per person per year during 1990–2006 and 2007–2010, respectively [21]. From 1990 to 2010, we did a longitudinal study involving most of the population of the village (all 247 inhabitants of the village in June 1990 at the beginning of the project, 468 of 509 inhabitants in December 2010).

The study area, the procedures of medical, parasitological, entomological and epidemiological surveillance and the main characteristics of malaria in this village have been described previously [21], [22]. Briefly, during 20 years, we visited all households daily, and collected nominative information on the presence or absence in the village of each individual we had enrolled, their location when absent, and the presence of fever or other symptoms. We systematically recorded body temperature at home three times a week (every second day except Sunday) in children younger than 5 years, and in older children and adults in case of suspected fever or fever-related symptoms. In cases of fever or other symptoms, blood testing was done by finger prick at our dispensary located in the village, and we provided detailed medical examination and specific treatment. The dispensary created for our project was open 24 h a day, 7 days a week, to allow both active and passive case detection. To investigate asymptomatic malaria carriage, we performed cross-sectional surveys at least quarterly in all individuals enrolled in the project. Blood was taken by finger prick and we examined 200 oil-immersion fields. We measured the parasite: leukocyte ratio for each plasmodial species and we enumerated separately the gametocytes of *P. falciparum*.

Between June 1990 and December 2010, four first-line drugs regimens were successively used for antimalarial treatment: oral quinine (Quinimax®) (October 1990–December 1994), chloroquine (January 1995–October 2003), sulfadoxine/pyrimethamine+amodiaquine (SP+AQ) (November 2003–May 2006) and artesunate+amodiaquine (AS+AQ) (June 2006–December 2010). Antimalarials were systematically given to young children in case of fever associated with a parasite: leukocyte ratio ≥2 [22]. When parasitemia was lower, the requirement for antimalarial treatment was decided taking into account all the patient's clinical, biological and epidemiological data [22]. Among older children (≥10 years) and adults permanently living in the village, it was rapidly observed that clinical malaria attacks lasted only a few hours even in cases where specific malaria treatment was delayed or not taken [23], and thus in most cases (except pregnant women) only symptomatic treatment was given under close clinical surveillance (three daily visit at home until recovery) in order to reduce the selection of drug resistant malaria parasites. Urine tests carried out to detect the presence of antimalarials indicated that almost all positive results (>99%) were explained by treatments given in our clinic [22]. There were no chemoprohylaxis, intermittent preventive treatment nor presumptive malaria treatment in children or adults during the time period 1990–2010. At the beginning of the project, 48.6% (children: 51.1%, adults: 47.1%) of the villagers used traditional mosquito nets, which were untreated, and this proportion remained almost unchanged until July 2008 when LLINs were distributed to all villagers.

### Definition of case, control and *P. falciparum* attack observations

A total of 64,262 simultaneous measurements of parasitemia and temperature made from June 1990 to December 2010 among 760 individuals aged from two weeks to 99 years were included in the analysis. The following definitions of case and control observations were used.

#### Case observations

Individual observations were regarded as fever cases if the rectal temperature measured by active case detection at home or by passive case detection at the clinic was ≥38°C (young children) or the axillary temperature was ≥37.5°C (older children and adults). Two fever episodes were considered independent if they occurred fifteen days apart or more. When several simultaneous measurements of parasitemia and temperature were available for the same fever episode, only the highest measure of parasitemia and the temperature associated were taken into account. 14,819 observations of parasitemia and temperature matched the case definition.

#### Control observations

Owing to the erratic nature of hyperthermia during malaria attacks and a number of other diseases, individual observations from the cross sectional surveys were considered to be asymptomatic controls if rectal/axillary temperatures were lower than 38°/37.5°C and if there was no episode of illness (allegation of fever and/or other fever related symptoms) between fifteen days prior and seven days after the temperature was taken. Measurements from pregnant women were excluded from the analysis. 49,443 observations of parasitemia and temperature collected during cross-sectional surveys matched the control definition.

#### *P. falciparum* attack observations

For incidence density calculation, *P. falciparum* clinical malaria attacks were defined as any case with fever or allegation of fever and/or fever-related symptoms (hot body, asthenia, cephalalgia, vomiting, diarrhea, abdominal pain, cough) whose parasitemia was higher than the age and period corresponding pyrogenic threshold derived from the model below. Cases were counted separately if they occurred fifteen days apart or more.

### Pyrogenic thresholds calculation

The pyrogenic thresholds were investigated separately at the beginning of the study (baseline period) and during the five successive treatment periods: the oral quinine period (excluding June–September 1990), the chloroquine period, the SP+AQ period, the ACT period (June 2006–July 2008) and the ACT+LLIN period (August 2008–December 2010). Data from the baseline period are those collected during the first four months of the study, from June to September 1990 [22]. Since patients with malaria attacks were also treated with oral quinine during this four months period, only individual observations collected up to the first treatment were included in the analysis for these patients.

The individual risk of hyperthermia was analyzed as a logistic regression of age and *P. falciparum* parasitemia. To take into account the interdependence of successive observations in the same individuals, a random-effect logistic regression model was used [7]. With this model, the estimated odds ratio can be considered as an estimation of the individual risk of fever associated with a variation in parasitemia. The correlation between observations was assumed to be induced by the existence of a person-cluster effect that was added to each logit in the person-cluster. The logit of the probability π_ij_ that the individual *i* presents a febrile episode during the observation *j* can be expressed in the form of a linear function of age z_i_ and parasite density x_ij_. Taking age into account, the best fit was obtained using a series of three dummy variables z_ik_ were k was an index coding for the four following age groups: 0–1, 2–6, 7–12, and ≥13 years old. Several different functions f(x_ij_) of parasitemia were fitted into the model. As previously observed [21], the *r*th power of x was the best function (i.e. according to the best log likelihood fit) for the parasitemia to describe the fever risk as a continuous function of parasite density. The exponent *r* for the function f(x_ij_) of parasitemia was tested at different values between 0.10 and 1.00 with a step of 0.05 for the first loop and then it was specified with a precision of 0.01. The existence of a threshold effect in addition to the previous continuous effect of parasitemia was demonstrated earlier [7] and was introduced as a binary variable s_ij_. It took the value 0 when the *j*th parasitemia of the individual *i* was below the tested threshold level and the value 1 when it was higher. β_0_ was a constant, α_i_ was the random-effects individual terms, β_1_, β_2_ and β_3_ were the regression coefficients:
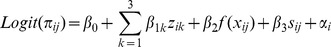



The models for each study period were compared according to the maximum likelihood (minimum deviance) using the Akaike method [24]. At each modeling step an individual-random effect was tested for a linear interaction of parasite density and age and period. All analyses were done with STATA software version 11.0 (College Station, TX, USA).

The pattern of the pyrogenic threshold was defined using five parameters: the age of maximal parasitemia in years (*a*), the highest parasitemia (*b*), parasitemia at age 0 (*c*), the level of parasitemia in oldest adults corresponding to a plateau (*d*), and finally a parameter determining the shape of the decrease of the function (*e*). Equation *h*_1_ represent threshold values in infants and younger children before age *a* (maximal parasitemia), and equation *h*_2_ represent threshold values in children and adults after age *a*.
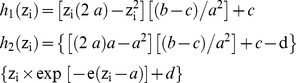



Parameter *e* was fixed at 0.09 according to preliminary analysis and the four other parameters were estimated for each period using the model by successive fitting of data, by varying them independently from each other, and by minimizing the Akaike criterion (AIC) [24]. Parameters *b* and *c* were estimated with a precision of ±500 trophozoites/µl and parameter *d* with an additional precision of ±200 trophozoites/µl for values <1000 trophozoites/µl.

All parasitemia estimates used in the models were derived from the parasite: leukocyte ratio that was measured when examining thick blood films. Since there was no simultaneous measurement of leukocytemia, when expressing the results in numbers of trophozoites per µl of blood, we adopted a mean standard leukocyte count of 8,000 per µl of blood for all age groups. This tends to underestimates the level of parasitemia in young children (average leukocyte counts ranging from 11,000 per µl in infants and about 10,000 per µl between two and four years), and to overestimates parasitemia in adults (average leukocyte counts between 5,000 and 6,500 per µl in African populations) [25].

### Ethical considerations

The project was initially approved by the Ministry of Health of Senegal and the assembled village population, and renewed on a yearly basis. Written informed consents were obtained individually from all participants or the parents of children younger than 15 years. For children participants aged 15–18 years, written informed consent were obtained individually since the National Ethics Committee of Senegal considered that participants in this age are responsible for their own person. Audits were regularly carried out by the National Ethics Committee of Senegal and ad-hoc committees of the Ministry of Health, the Pasteur Institute (Dakar, Senegal), and the Institut de Recherche pour le Développement (formerly ORSTOM, Paris and Marseille).

## Results

### Asymptomatic *P. falciparum* infections

**Figure 1:**
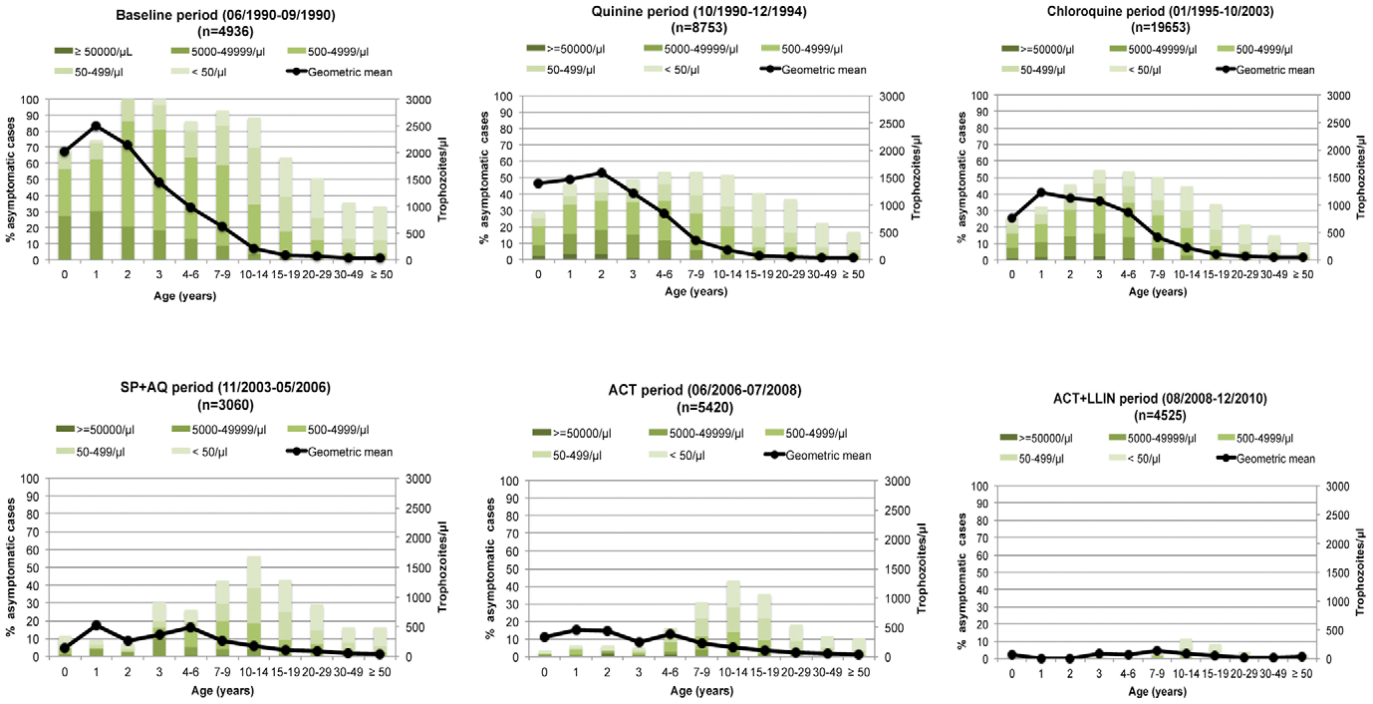
Age distribution of parasite rate, classes of parasite density and the mean *P. falciparum* asymptomatic parasitemia (geometric mean of trophozoites per µl of blood) in control observations for each study period.

*P. falciparum* malaria prevalence and the mean level of parasitemia during asymptomatic infections decreased markedly from 1990 to 2010 (Fig. 1). During the baseline period, the proportion of asymptomatic control observations with *P. falciparum* trophozoïtes was 70% in infants and reached 100% in children 2 and 3 years of age. During the most recent period, it was only 1% in these children. In adults ≥50 years, malaria prevalence declined from 34% to 2% between 1990 and 2010. In children, there was also a marked decrease of the mean asymptomatic parasitemia, from ≈2,000–2,500 trophozoites per µl of blood in children 0–3 years during the baseline period to less than 200 trophozoites per µl in these children during the ACT+LLINs period. In older children and adults, the mean asymptomatic parasitemia was already very low at the beginning of the project, ranging between 50 and 200 trophozoites per µl, and the decrease was less marked. As shown in Fig. 1, the decrease of *P. falciparum* prevalence and asymptomatic parasite density in children occurred by steps, a first step corresponding to the first treatment period (quinine) and a second step when SP+AQ replaced chloroquine. In adults, prevalence decreased markedly after the additional introduction of LLINs in the last period.

### *P. falciparum* parasitemia during fever episodes

**Figure 2:**
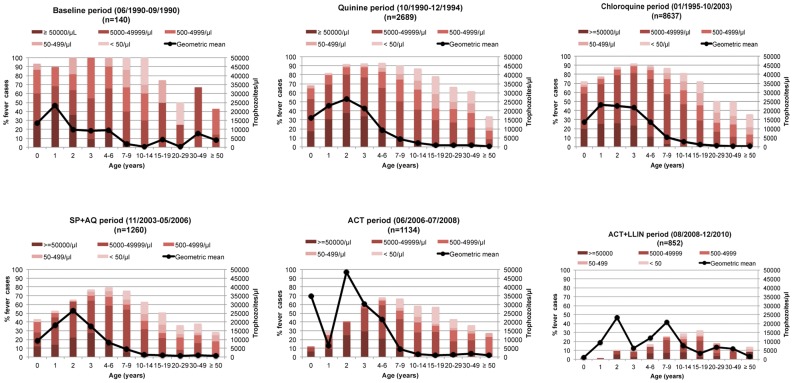
Age distribution of parasite rate, classes of parasite density and the mean *P. falciparum* parasitemia observed during all causes of fever episodes (geometric mean of trophozoites per µl of blood) for each study period.

For each period and age group, the proportion of individuals with fever harbouring *P. falciparum* malaria parasites and the mean level of parasitemia during these infections are shown in Fig. 2. In children, ≈90–100% of fever cases were associated with malaria infections during the baseline period. This proportion remained high (≈70–90%) during the quinine and chloroquine periods, but declined to much lower levels in younger children during the following periods. Only 2% of fever cases were associated with malaria parasites in children <2 years during the ACT+LLINs period. During each period, the highest levels of parasitemia were observed in younger children and there was no clear change, according to periods, of mean *P. falciparum* levels during fever episodes.

### Attributing fever episodes to *P. falciparum* malaria

Estimates with the lowest deviance of the parameters defining the shape and level by age of pyrogenic thresholds during the baseline and the five treatment periods are given in Table 1. Highest threshold parasitemia decreased from 21,500 trophozoites per µl of blood in children one year of age during the baseline period to 10,000 trophozoites per µl of blood in children two years of age during the ACT+LLINs period. Lowest threshold parasitemia in adults decreased from 2,500 to 500 trophozoites per µl of blood between these two periods.

**Table 1:** Estimates of the parameters defining the age-dependent pyrogenic threshold according to study period.

	Baseline period (06/1990–09/1990)	Quinine period (10/1990–12/1994)	Chloroquine period (01/1995–10/2003)	SP+AQ period (11/2003–05/2006)	ACT period (06/2006–07/2008)	ACT+LLINs period (08/2008–12/2010)
**Number of individuals**	200	386	518	404	479	482
**Number of case observations**	140	2,689	8,637	1,260	1,134	852
**Number of control observations**	4,936	8,753	19,653	3,060	5,420	4,525
**Exponent for the function of parasitemia (** ***r*** **)**	0.82	0.31	0.37	0.58	0.43	0.72
**Age in years of maximum parasitemia (** ***a*** **)**	1	2	2	2	2	2
**Maximum threshold parasitemia (** ***b*** **)**	21,500	16,000	14,000	14,000	14,000	10,000
**Parasitemia at year 0 (** ***c*** **)**	20,500	15,000	12,000	3,000	2,500	2,500
**Lowest threshold parasitemia in adults (** ***d*** **)**	2,000	1,500	1,500	1,000	500	500
**Threshold effect odds ratios (95% CI)**	33.48 (11.82–94.82)	2.72 (2.11–3.51)	3.01 (2.58–3.53)	2.95 (1.68–5.17)	4.28 (2.55–7.17)	8.49 (1.12–64.08)
**Continuous effect of parasitemia odds ratios for an increase of 100 trophozoites/µl (95% CI)**	1.04 (1.04–1.05)	1.90 (1.84–1.96)	1.61 (1.59–1.64)	1.18 (1.16–1.20)	1.39 (1.35–1.43)	1.17 (1.12–1.23)
**0–1 year old effect odds ratios**	1.00	1.00	1.00	1.00	1.00	1.00
**2–6 years old effect odds ratios (95% CI)**	0.52 (0.28–0.98)	0.73 (0.59–0.90)	1.21 (1.07–1.36)	1.42 (1.05–1.93)	0.62 (0.49–0.79)	1.01 (0.80–1.28)
**7–12 years old effect odds ratios (95% CI)**	0.24 (0.11–0.49)	0.81 (0.63–1.04)	1.55 (1.33–1.79)	0.66 (0.47–0.91)	0.34 (0.26–0.45)	0.62 (0.46–0.82)
**≥13 years old effect odds ratios (95% CI)**	0.06 (0.03–0.13)	0.49 (0.39–0.61)	1.05 (0.90–1.22)	0.40 (0.29–0.54)	0.18 (0.14–0.23)	0.36 (0.28–0.46)
**AIC**	780.55	8476.45	23751.08	3792.72	4516.79	4263.92

Since threshold levels decreased more markedly between the baseline period and the quinine period, then between the chloroquine and the SP+AQ periods, we investigated whether these decreases were progressive or occurred abruptly. When modelling data of the period October 1990–March 1991, i.e. the first six months of the quinine period, we obtained values of the parameters *r*, *a*, *b*, *c* and *d* very close or similar to those of the whole period. Similar results were also obtained when comparing data of the period December 2003–June 2004, i.e. the first six months of the SP+AQ period, with those of this whole period (data not shown).

**Figure 3:**
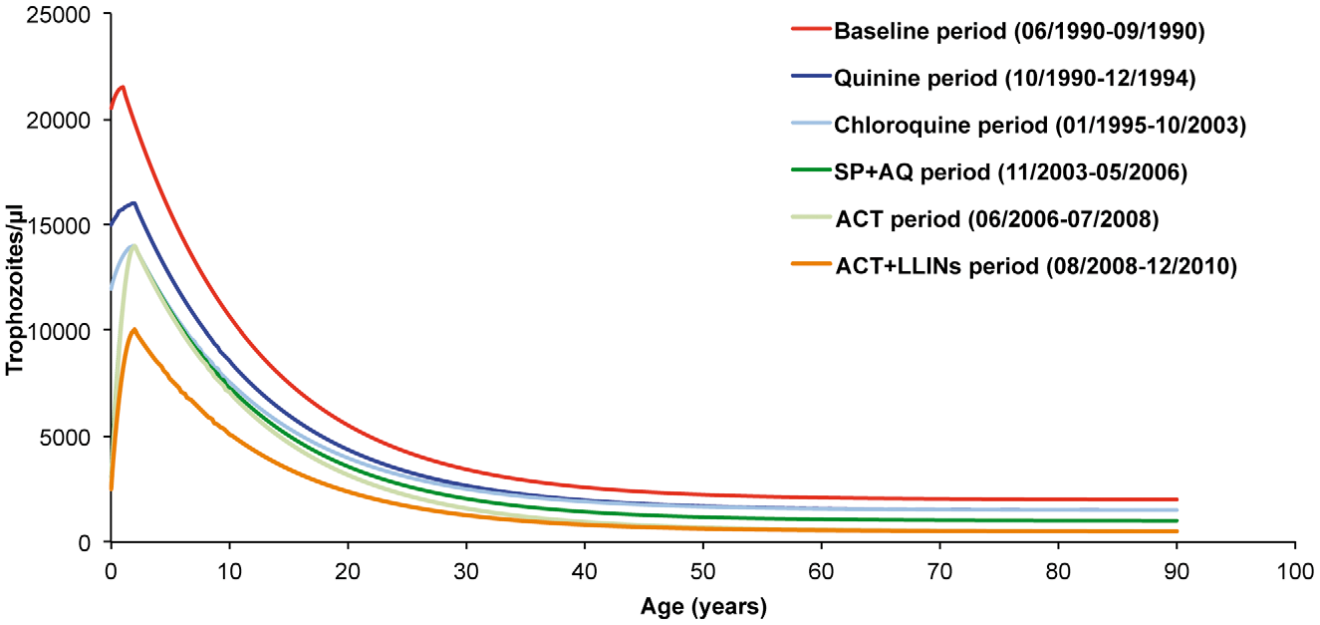
Random-effect logistic regression model derived threshold levels of parasitemia for attributing fever episodes to *P. falciparum* malaria by age and periods.

Fig. 3 shows the level of the pyrogenic thresholds by age and periods. During the baseline period, 23 of 4,936 measurements of parasite density in asymptomatic individuals (0.5%) and 62 of 140 measurements in fever cases (44.3%) were above the pyrogenic threshold. The number of measurements above the parasite threshold in asymptomatic individuals was 264/8,753 (3.0%), 620/19,653 (3.1%), 58/3,060 (1.9%), 86/5,420 (1.4%) and 4/4,525 (0.1%) during the quinine, chloroquine, SP+AQ, ACT, and ACT+LLINs periods, respectively. The number of measurements above the parasite threshold in fever cases was 1,352/2,689 (50.3%), 4,476/8,637 (51.8%), 523/1,260 (41.5%), 405/1,134 (35.7%) and 87/852 (10.2%) during the quinine, chloroquine, SP+AQ, ACT, and ACT+LLINs periods, respectively.

We compared three definitions of malaria attacks: (A) fever cases with parasitemia higher than the threshold measured for the corresponding treatment period; (B) fever cases with parasitemia higher than the baseline period threshold; and (C) all fever cases associated with the presence of malaria parasites, whatever the level of parasitemia. Table 2 shows that considering as malaria attacks all fever cases associated with the presence of malaria parasites overestimates considerably the number of clinical malaria attacks in all age groups and in all periods. Overestimation was greatest during the quinine period (68.5% overestimation in children, 211.7% overestimations in adults, 86.7% overall), and lowest during the ACT+LLINs period (42.2% in children, 45.9% in adults, 43.6% overall). When thresholds of the baseline period were used during treatment periods instead of period-specific thresholds, the numbers of clinical malaria attacks were underestimated by 9.8–20.2% in children and 18.9–40.2% in adults according to treatment periods (Table 2).

**Table 2:** Number of *P. falciparum* malaria attacks by treatment period and age according to three definitions of malaria attacks: A. Fever or fever-related symptoms plus parasitemia higher than the period specific threshold level.

		Age (in years)										
Periods	Malaria definitions	0	1	2	3	4–6	7–14	15–29	≥30	TOTAL (0–14)	TOTAL (≥15)	TOTAL (All ages)
**Quinine**	**A**	**103**	**192**	**244**	**193**	**347**	**267**	**118**	**78**	**1,346**	**196**	**1,542**
	**B**	88 (−14.6%)	169 (−12.0%)	230 (−5.7%)	183 (−5.2%)	319 (−8.1%)	225 (−15.7%)	99 (−16.1%)	49 (−37.2%)	1,214 (−9.8%)	148 (−24.5%)	1,362 (−11.7%)
	**C**	170 (+65.0%)	278 (+44.8%)	320 (+31.1%)	266 (+37.8%)	595 (+71.5%)	639 (+139.3%)	390 (+230.5%)	221 (+183.3%)	2,268 (+68.5%)	611 (+211.7%)	2,879 (+86.7%)
**Chloroquine**	**A**	**286**	**476**	**647**	**619**	**1,425**	**1,169**	**298**	**233**	**4,622**	**531**	**5,153**
	**B**	231 (−19.2%)	403 (−15.3%)	561 (−13.3%)	535 (−13.6%)	1,134 (−20.4%)	873 (−25.3%)	201 (−32.5%)	124 (−46.8%)	3,737 (−19.1%)	325 (−38.8%)	4,062 (−21.2%)
	**C**	457 (+59.8%)	627 (+31.7%)	841 (+30.0%)	803 (+29.7%)	2,081 (+46.0%)	2,441 (+108.8%)	981 (+229.2%)	693 (+197.4%)	7,250 (+56.9%)	1,674 (+215.2%)	8,924 (+73.2%)
**SP+AQ**	**A**	**27**	**50**	**67**	**80**	**192**	**184**	**34**	**41**	**600**	**75**	**675**
	**B**	19 (−29.6%)	42 (−16.0%)	59 (−11.9%)	67 (−16.2%)	157 (−18.2%)	135 (−26.6%)	21 (−38.2%)	26 (−36.6%)	479 (−20.2%)	47 (−37.3%)	526 (−22.1%)
	**C**	45 (+66.7%)	70 (+40.0%)	83 (+23.9%)	105 (+31.2%)	311 (+62.0%)	384 (+108.7%)	112 (+229.4%)	85 (+107.3%)	998 (+66.3%)	197 (+162.7%)	1,195 (+77.0%)
**ACT**	**A**	**9**	**19**	**43**	**56**	**150**	**148**	**46**	**36**	**425**	**82**	**507**
	**B**	8 (−11.1%)	16 (−15.8%)	41 (−4.6%)	48 (−14.3%)	139 (−7.3%)	115 (−22.3%)	27 (−41.3%)	22 (−38.9%)	367 (−13.6%)	49 (−40.2%)	416 (−17.9%)
	**C**	12 (+33.3%)	35 (+84.2%)	53 (+23.2%)	67 (+19.6%)	192 (+28.0%)	282 (+90.5%)	112 (+143.5%)	70 (+94.4%)	641 (+50.8%)	182 (+121.9%)	823 (+62.3%)
**ACT+LLINs**	**A**	**0**	**0**	**5**	**5**	**15**	**39**	**22**	**15**	**64**	**37**	**101**
	**B**	0 (−0.0%)	0 (−0.0%)	5 (−0.0%)	2 (−60.0%)	13 (−13.3%)	32 (−17.9%)	17 (−22.7%)	13 (−13.3%)	52 (−18.7%)	30 (−18.9%)	82 (−18.8%)
	**C**	1	1	6 (+20.0%)	7 (+40.0%)	19 (+26.7%)	57 (+46.1%)	33 (+50.0%)	21 (+40.0%)	91 (+42.2%)	54 (+45.9%)	145 (+43.6%)

B. Fever or fever-related symptoms plus parasitemia higher than the baseline threshold. C. Fever or fever-related symptoms plus any level of parasitemia. Values in bracket with definition B indicate the proportion of underdiagnosed malaria attacks compared to definition A. Values in bracket with definition C indicate the proportion of overdiagnosed malaria attacks compared to definition A. Dielmo, October 1990–December 2010.

## Discussion

Because of the high prevalence of asymptomatic malaria infections and the non-specific signs and symptoms of the disease, the diagnosis of clinical malaria presents difficult methodological problems in malaria endemic areas, critical for first-line treatment decision and for accurate evaluation of any intervention aimed at malaria morbidity reduction. To estimate the fraction of fever cases that are attributable to malaria, several approaches corresponding to different definitions of malaria morbidity have been used in the literature, either for establishing national vital statistics or as part of specific studies. In most health facilities in Africa, there is no laboratory test for confirming or discarding the clinical diagnosis of malaria, and any fever case is presumed to be a malaria attack when specific signs of other diseases are lacking [26]. Published national and international statistics of malaria morbidity and mortality are thus essentially based on presumed cases of malaria, although many studies have clearly demonstrated the poor value of clinical criteria for distinguishing malaria from other causes of fever [1], [27]–[29]. Recently, the deployment of ACTs in Senegal and in other African countries has often been associated with the implementation of rapid diagnostic tests (RDT) for malaria, resulting in a dramatic decrease in the number of cases attributed to malaria in health facilities and raising new issues about the respective role of ACTs and RDTs in the reported decrease of malaria [21], [30]. Our data clearly show that new malaria control policies dramatically reduced the burden of malaria whatever the criteria used for defining clinical malaria.

Since the mid-1980s, using various approaches (e.g. direct comparisons of parasitemia levels, attributable fractions, case control studies, or models testing an age-dependent threshold effect), the parasite density method has become in Africa the gold standard to assess morbidity from malaria in community or out-patient studies, and to measure the efficacy of control strategies such insecticide-treated nets, drug prevention protocols, or malaria vaccine candidates [1]–[14]. Parasite density measurement proved also very useful to guide therapeutic decisions, since the presumptive malaria treatment of febrile patients often delays the specific treatment of other diseases, in particular the antibiotic treatment of bacterial diseases that may represent a frequent cause of morbidity [31]. Our results show that under all conditions of endemicity, defining malaria attacks as all fever cases associated with any level of parasitemia dramatically overestimated the burden of malaria in all age groups. This was expected under conditions of high endemicity, but our study also shows that this definition of malaria attacks also overestimates markedly the burden of malaria under hypoendemic conditions, both among children and adults. Interestingly, it is not clear whether it may be some situations where any parasitemia in a febrile patient is likely to represent febrile malaria, since the more parasite rates are decreasing in the community, the more long-lasting asexual parasitemias are needed to induce the production of gametocytes that are short lived (maximum 3–4 weeks [32]) but necessary to maintain at least low levels of malaria transmission.

To determine the level of the threshold of parasitemia for diagnosing malaria attacks, many studies have used the attributable fractions method, either to select threshold levels presenting the best compromise between sensitivity and specificity, or to select thresholds levels giving estimates of malaria incidence close of those directly calculated by the attributable fraction method. The concept of pyrogenic threshold is an old concept in malarialogy [33], [34], and our findings in Dielmo show that the relationship between parasite density and fever risk is much better described as an age-dependent discontinuous function than a continuous one. This was clearly the case during the baseline period, where the subject-specific fever risk was multiplied by an odds ratio of 33 when a person's parasitemia crossed the threshold level corresponding to his or her age, but this was also observed with lower odds ratio during each treatment period, including the most recent one where malaria prevalence decreased to very low levels in each age group. According to treatment period, differences between estimates of malaria morbidity at the community level obtained by the attributable fraction method compared to those obtained by the threshold method ranged from+12.3% (quinine period) to −6.1% (SP+AQ period).

Data collected in Dielmo during 20 years show that even a limited use of antimalarials, strictly restricted to parasitologically confirmed malaria attacks, may have a major impact on asymptomatic parasite carriage. As expected, the decrease of parasitemia in asymptomatic individuals significantly decreased the threshold levels of parasitemia to be used for the assessment of malaria morbidity, with the lowest thresholds levels during the ACT+LLINs period. Interestingly, although using baseline threshold levels would underestimate the burden of malaria in all age-groups and in all treatment periods, differences between baseline period and period-specific based estimates of malaria morbidity only range between 11.7% and 22.1% at the community level, while the decrease of threshold levels was at least halved between baseline and the last periods. In fact, most malaria attacks in children are associated with peaks of parasite density much higher than the range of threshold levels derived from the models, and only a low proportion of asymptomatic parasitemia were close to this range. In adults, due to strong protective immunity, the duration of the peaks of parasitemia responsible for fever and other symptoms only last a few hours, and patients were seen in the dispensary at various stages of their clinical episode, often when parasitemia was probably yet decreasing [23].

Our study shows that malaria control modifies in all age-groups the threshold levels of parasitemia to be used for the assessment of malaria morbidity. Although treating with antimalarials any parasitologically confirmed infection may often be justified in order to prevent any risk related to misdiagnosis and/or to long-lasting parasite carriage in terms of morbidity and transmission, it appears that even under the low levels of malaria endemicity, where most young children are non-immune and acquired immunity in other age groups is declining, the parasite density method must remain in Africa the gold standard for distinguishing malaria from other causes of fever and assessing trends in the burden of malaria.
